# Triterpene Constituents of *Euphorbia Erythradenia *Bioss. and their Anti-HIV Activity

**Published:** 2016

**Authors:** Abdul Majid Ayatollahi, Seyed Mohammad Zarei, Arash Memarnejadian, Mustafa Ghanadian, Mohammad Heydarian Moghadam, Farzad Kobarfard

**Affiliations:** a *Phytochemistry Research Center and School of Pharmacy, Shahid Beheshti University of Medical Sciences, Tehran, Iran. *; b *Department of hepatitis and AIDS, Institute Pasture of Iran, Tehran, Iran. *; c *Department of Pharmacognosy, Isfahan Faculty of Pharmacy, Isfahan University of Medical Sciences, Isfahan, Iran. *; d *Department of Audiology, school of Rehabilitation, Shahid Beheshti University of Medical Sciences, Tehran, Iran. *; e *Department of Medicinal chemistry, School of Pharmacy, Shahid Beheshti University of Medical Sciences, Tehran, Iran.*

**Keywords:** Euphorbia erythradenia, triterpene, NMR, anti HIV, docking

## Abstract

Phytochemical investigation of the aerial parts of *Euphorbia erythradenia* Bioss. (Euphorbiaceae), one of Iranian endemic *Euphorbia*s, with particular attention to triterpene constituents, using methanol solvent extraction was carried out. Five known triterpenes, including four cycloartanes and oleanolic acid, were isolated for the first time and identified using NMR and Mass techniques. Anti HIV activity of the isolated triterpenes and ingenoid diterpenes was evaluated using single cycle replicable HIV-1 (SCR HIV-1) virions. Molecular features of the most active compound (IC_50_ = 0.008 μM, CC_50_ = 3.264 μM, TI = 380.64), which showed higher therapeutic index than nevirapine, was assessed using molecular docking. Docking studies demonstrated three hydrogen bonds between HIV-1 virion protease active site and this compound with a distance less than 3 A° which can be responsible for the observed anti HIV-1 activity.

## Introduction

The genus *Euphorbia* is the largest among the plant family Euphorbiaceae, comprising about 2000 known species consisting of annuals to trees. They all contain latex and have unique flower structures. About 70 species have been reported in Iran that 17 of them are endemic (1). *E. erythradenia *Bioss. is one of Iranian endemic spurges (2) which few phytochemical studies have been reported for this plant. Some species of this genus are used in folk medicines to cure skin diseases, gonorrhea, migraines, intestinal parasites, and warts (1) which most of them have been proved by modern phytochemical and pharmacological studies. In Iran, they are also used as a purgative.

The presence of several kinds of secondary metabolites including triterpenes, steroids, flavonoids, phenolics and aromatic compounds in *Euphorbia* species have been reported up until now (1, 3), but what highlights the features of *Euphorbia*’s secondary metabolites, is its specific terpenoids profile. *Euphorbia*’s terpenoids, including diterpenes and triterpenes, have shown interesting various biological activities which are attracting medicinal researchers increasingly as a source of new potential drugs as well as lead compounds.

**Figure 1:**
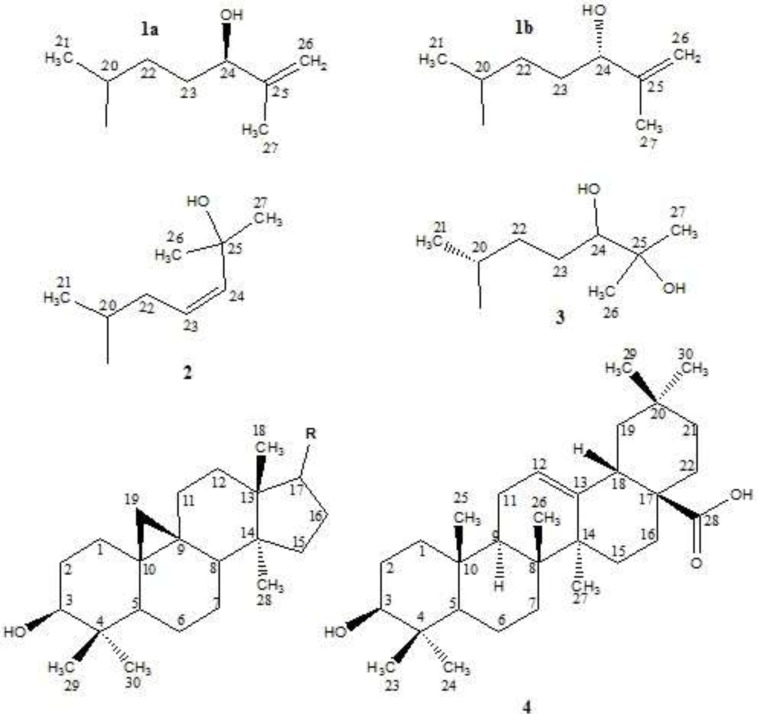
Isolated triterpenes from *E. erythradenia*. (24*R*)-Cycloart-25-ene-3*β*,24-diol (1a); (24*S*)-Cycloart-25-ene-3*β*,24-diol(1b); Cycloart-23-ene-3*β*,25-diol(2); Cycloart-3*β*,24,25-triol(3); Oleanolic acid (4).

**Figure 2:**
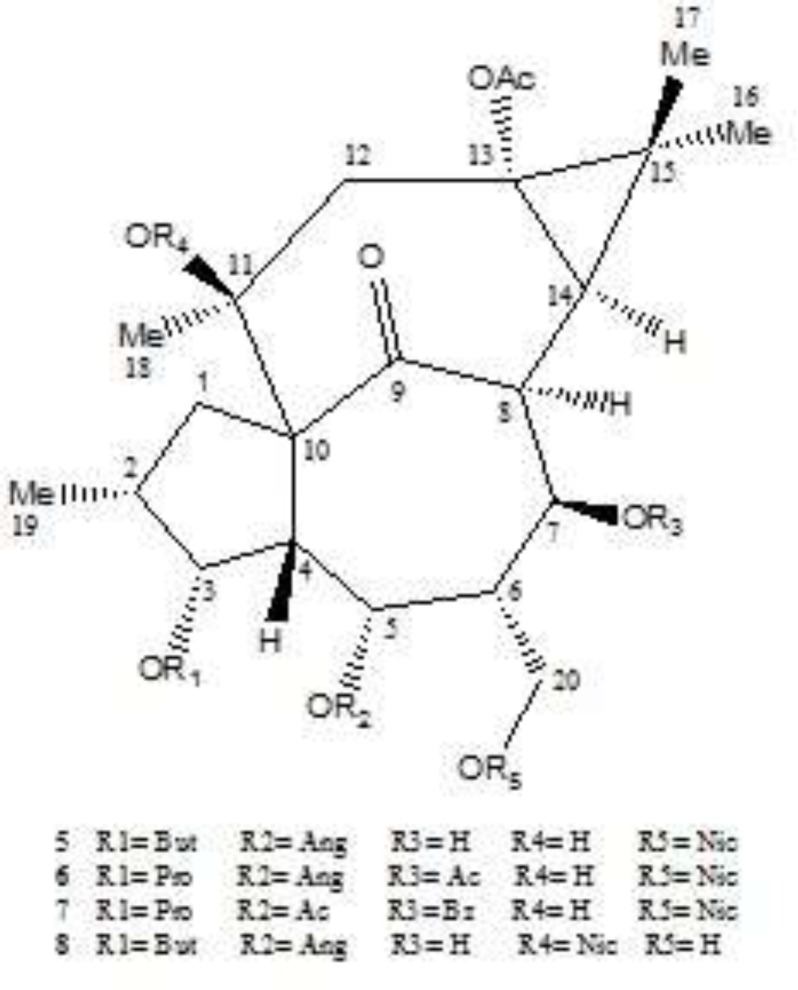
Isolated diterpenes from *E. erythradenia*. 13-acetyl-3-butanoyl-5-angeloyl-20-nicotinyl-1,2,6,7-tetrahydroingenol (5); 7,13-diacetyl-5-angeloyl-20-nicotinyl-3-propionyl-1,2,6,7-tetrahydroingenol (6); 5,13-diacetyl-7-benzoyl-20-nicotinyl-3-propionyl-1,2,6,7-tetrahydroingenol (7); 13-acetyl-5-angeloyl-11-nicotinyl-3-butanoyl-1,2,6,7-tetrahydroingenol (8).

In this paper, as a part of our efforts to find new sources of bioactive terpenoids, one of Iranian *Euphorbia* species, *E. erythradenia* Aerial parts were phytochemically investigated and five known triterpene compounds extracted and their chemical structure were elucidated (Figure 1). Considering the huge demand for finding new drugs for viral infections, specially acquired human immunodeficiency virus (HIV), anti HIV activity of the extracted triterpenoids plus the four previously reported ingenoid diterpenes from *E. erythradenia* (Figure 2), were evaluated, using single cycle replicable HIV virions. In order to find the important structural features of anti HIV-1 active compounds, ligand-protein binding studies were carried out using AutoDock software.

## Experimental

*General*

Column chromatography (CC): silica gel 63–200 μm; TLC: silica gel GF_254_ plates (20×20 cm, 0.5 mm, Merck); detection by spraying with cerium sulphate in 10% aq. H_2_SO_4_ and heating. Preparative HPLC: Agilent 1100 Series with a normal phase column (250×20 mm i.d.) packed with 5µm silica (YMC Co., Ltd., Kyoto, Japan) that was equipped with 515 Waters pump and UV-Vis detector. All solvents used were of HPLC grade (Caledon Laboratory Chemicals Ltd. Georgetown, Ont. Canada). NMR: Bruker AV-400 (^1^H) and AV-100 (^13^C), *δ* in ppm rel. to Me4Si and *J* in Hz; HR-ESI-MS: Waters Q-TOF Micro YA019 mass spectrometer in m/z and EI-MS spectra: Varian MAT 112 or MAT 312 spectrometers.

*Plant material*

Aerial flowering parts of *E. erythradenia* f. (Euphorbiaceae) were collected in September 2010 from populations growing in Gharbalbiz in the neighborhood of Mehriz city, Yazd province (I.R.Iran). Plant material was identified by Ali Mirhosseini, plant taxonomist (Agricultural and natural Resources Research Center of Yazd Province) and a voucher specimen (nos. 1947) was deposited in the Herbarium Center of Yazd (HCY).

*Extraction and isolation*

The air-dried powdered plant (2.8 kg) was macerated for four days with methanol (9 L×3), at room temperature. Filtration and under vacuum evaporation resulted in a green gum (433.2 g, 15.47% of dried powder weight), that was suspended in 1 liter methanol 80% and fractionated with hexane (1 lit × 3) which led to 51.3 g (1.83% of dried powder weight) hexane fraction. Methanolic suspension was further fractionated three times with 1 liter of chloroform that resulted in 37.7g chloroform fraction (1.35% of dried powder weight). Chloroform fraction was subjected to silica gel column, using hexane/chloroform (30→100) and chloroform/methanol (0→20), to render several fractions: F_1-4_. Subsequently, the richest fraction in triterpenoids, F_2_ (based on TLC visualization after cerium sulfate solvent spraying and heating), was chromatographed on silica gel C.C. (hexane/ethyl acetate, 0→60) to afford five subfractions; TF_2a_-TF_2e_. Subfractions were injected into preparative HPLC using hexane/ethyl acetate gradient system at detection wavelength of 270 nm. Fractions were inspected and combined using TLC and heating after acidic cerium sulfate solvent spraying which resulted into pure compounds: TF_2b1_ (1a & 1b, 15 mg), TF_2c2_ (2, 18 mg), TF_2d5_ (3, 13mg). Moreover F_3_ was subjected to column of silica gel and washed with hexane/ethyl acetate (0→20). Then TF_3.101-103 _subfractions were combined and recrystallized with cold chloroform which resulted to compound TF_3.101_ (4, 26 mg). 

*(24R)-Cycloart-25-ene-3β,24-diol (1a)*

White, amorphous powder; 15 mg (1a & 1b);C_30_H_50_O_2_; MW (g/mol): 442; yield: 0.00054%; EI-MS m/z (rel. int.): 52 (47.7), 69 (42.6), 80 (100), 98 (46.2), 107 (27.4), 121 (19.6), 135 (16.3), 147 (14.9), 161 (11.4), 175 (14.9), 187 (7.0), 203 (8.8), 227 (4.2), 269 (3.1), 297 (3.5), 302 (8.6), 315 (2.9), 325 (1.1), 355 (2.2), 381 (3.7), 409 (6.2), 424 (4.6), 442 (2.0); ^1^H-NMR data (400 MHz, CDCl_3_, J in Hz ) see Table 1; ^13^C-NMR data (100 MHz, CDCl_3_) see Table 2.

**Table 1:** ^1^H-NMR (400 MHz, CDCl_3_, *δ*_H_ in ppm,* J* in Hz) data of *E. erythradenia* triterpenoids

***Pos***	**1a**	**1b**	**2**	**3**	**4**
*3*	3.28 *dd* (4.4, 10.8)	3.28 *dd* (4.4, 10.8)	3.28 *m*	3.28 *m*	3.22 *dd* (11.0, 4.6)
*12*					5.28 *t* (3.4)
*18*	0.96 *s*	0.96 *s*	0.96 *s*	0.97 *s*	2.82 *dd* (13.8, 4.2)
*19*	0.32 *d* (4.0)	0.32 *d* (4.0)	0.33 *d* (4.0)	0.33 *d* (4.0)	
	0.55 *d* (4.0)	0.55 *d* (4.0)	0.55 *d* (4.0)	0.55 *d *(4.0)	
*21*	0.88 *d* (5.6)	0.88 *d* (5.6)	0.86 *d* (6.8)	0.89 *d* (5.6)	
*23*			5.60 *m*		1.13 *s*
*24*	4.02 *t* (6.4)	4.02 *t* (6.4)	5.60 *m*	3.30 *m*	0.91 *s*
*25*	-	-	-	-	0.75 *s*
*26*	4.83 *br s* 4.93 *br s*	4.83 *br s* 4.93 *br s*	1.31 *s*	1.16 *s*	0.93 *s*
*27*	1.72 *s*	1.72 *s*	1.31*s*	1.22 *s*	0.99 *s*
*28*	0.89 *s*	0.89 *s*	0.89 *s*	0.89 *s*	
*29*	0.96 *s*	0.96 *s*	0.96 *s*	0.97 *s*	0.77 *s*
*30*	0.80 *s*	0.80 *s*	0.81 *s*	0.81 *s*	0.90 *s*

*(24S)-Cycloart-25-ene-3β,24-diol(1b):*

White, amorphous powder; 15 mg (1a & 1b); C_30_H_50_O_2_; MW (g/mol): 442; yield: 0.00054%; EI-MS *m/z* (rel. int.): 52 (47.7), 69 (42.6), 80 (100), 98 (46.2), 107 (27.4), 121 (19.6), 135 (16.3), 147 (14.9), 161 (11.4), 175 (14.9), 187 (7.0), 203 (8.8), 227 (4.2), 269 (3.1), 297 (3.5), 302 (8.6), 315 (2.9), 325 (1.1), 355 (2.2), 381 (3.7), 409 (6.2), 424 (4.6), 442 (2.0);^ 1^H-NMR data (400 MHz, CDCl_3_, *J* in Hz) see Table 1; ^13^C-NMR data (100 MHz, CDCl_3_) see Table 2.

**Table 2:** ^13^C-NMR (100 MHz, CDCl_3_, *δ*_C_ in ppm) data of *E. erythradenia* triterpenoids

***Pos***	**1a**	**1b**	**2**	**3**	**4**
*1*	31.9	31.9	32.0	32.0	38.4
*2*	30.4	30.4	30.4	30.4	27.2
*3*	78.8	78.8	78.8	78.8	79.0
*4*	40.5	40.5	40.5	40.5	38.8
*5*	47.1	47.1	47.1	47.1	55.2
*6*	21.1	21.1	21.1	21.1	18.3
*7*	28.1	28.1	28.1	28.1	32.6
*8*	48.0	48.0	48.0	48.0	39.3
*9*	20.0	20.0	20.0	20.0	47.6
*10*	26.0	26.0	26.0	26.1	37.1
*11*	26.0	26.0	26.0	26.0	22.9
*12*	32.9	32.9	35.6	32.9	122.6
*13*	45.3	45.3	45.3	45.3	143.6
*14*	48.8	48.8	48.8	48.8	41.0
*15*	35.5	35.5	32.8	35.5	27.7
*16*	26.4	26.4	26.4	26.5	23.4
*17*	52.1	52.1	52.0	52.3	46.5
*18*	18.0	18.0	18.1	18.1	41.6
*19*	29.9	29.9	30.0	29.9	45.9
*20*	35.9	35.9	36.4	36.4	30.7
*21*	18.3	18.3	18.3	18.4	33.8
*22*	31.9	31.9	39.0	33.5	32.4
*23*	31.5	31.6	139.3	28.7	28.1
*24*	76.3	76.7	125.6	79.6	15.6
*25*	147.5	147.7	70.7	76.2	15.3
*26*	111.4	110.9	29.9	23.2	17.0
*27*	17.2	17.6	29.9	26.5	25.9
*28*	19.3	19.3	19.3	19.3	183.3
*29*	14.0	14.0	14.0	14.0	33.1
*30*	25.4	25.4	25.4	25.4	23.6

*Cycloart-23-ene-3β,25-diol (* ***2*** *)*

White, amorphous powder; 18 mg; C_30_H_50_O_2_; MW (g/mol): 442; yield: 0.00064%; EI-MS *m/z* (rel. int.): 55 (73.2), 69 (78.2), 81 (79.1), 95 (98.0), 109 (100), 121 (72.3), 135 (55.6), 147 (62.0), 161 (48.8), 175 (45.9), 187 (39.8), 203 (45.7), 215 (18.0), 229 (14.3), 241 (11.4), 255 (31.0), 269 (17.8), 284 (11.4), 297 (10.1), 302 (26.6), 315 (7.3), 327 (11.9), 343 (7.3), 363 (4.4), 381 (12.7), 392 (12.3), 407 (4.6), 410 (32.7), 425 (22.4), 442 (6.2); ^1^H-NMR data (400 MHz, CDCl_3_, *J* in Hz) see Table 1; ^13^C-NMR data (100 MHz, CDCl_3_) see Table 2.

*Cycloart-3β,24,25-triol (* ***3*** *)*

Pale yellow, amorphous powder; 13mg; C_30_H_52_O_3_; MW (g/mol): 460; yield: 0.00046%; EI-MS *m/z* (rel. int.): 59 (78.0), 71 (100), 81 (61.5), 95 (85.3), 109 (69.9), 121 (51.6), 133 (49.2), 147 (38.5), 163 (45.9), 175 (50.5), 187 (21.8), 203 (31.9), 215 (11.0), 221 (22.0), 257 (8.6), 269 (6.2), 287 (8.4), 297 (11.0), 315 (13.2), 320 (21.8), 343 (1.1), 355 (4.0), 399 (7.9), 409 (9.0), 427 (10.8), 442 (11.9), 460 (4.4); ^1^H-NMR data (400 MHz, CDCl_3_, *J* in Hz) see Table 1; ^13^C-NMR data (100 MHz, CDCl_3_) see Table 2.

*Oleanolic acid (4)*

White, crystalline powder; 26 mg; C_30_H_48_O_3_; MW (g/mol): 456; yield: 0.00093%; EI-MS *m/z* (rel. int.): 55 (15.8), 69 (28.1), 81 (22.0), 85 (6.8), 95 (18.0), 105 (16.7), 119 (15.8), 133 (18.9), 147 (9.2), 163 (18.0), 175 (14.7), 189 (19.3), 203 (72.5), 207 (20.2), 233 (9.2), 248 (100), 300 (2.2), 411 (2.4), 439 (2.2), 456 (2.0); ^1^H-NMR data (400 MHz, CDCl_3_, *J* in Hz) see Table 1; ^13^C-NMR (100 MHz, CDCl_3_) see Table 2. HREI-MS *m/z* 457.3623 (Calcd. for C_30_H_48_O_3_ + H^+^).

*Production of Pseudotyped Single Cycle Replicable HIV Virions*

Single Cycle Replicable HIV-1 virions (SCR) were previously constructed and their characterization ascertained the production of infective HIV-1 virions with the capability of one cycle of replication (4, 5). Briefly, these single cycle replicable HIV-1 (SCR HIV-1) virions were produced by deleting a 2-kb fragment within the Pol region of the HIV-1 genome from the pNL4-3 strain. Pseudotyped SCR HIV-1 virions were constructed by co-transfection of HEK 293T cells with pmzNL4-3 (containing deleted genome), psPAX2, and pMD2G plasmids obtained from Addgene (www.addgene.org). The pmzNL4-3 plasmid encodes the HIV-1 full-length RNA, with packaging ability containing the aforementioned deletion in the Pol region; the psPAX2 plasmid encodes HIV Gag and Gag-Pro-Pol polyproteins, besides all the HIV-1 accessory proteins; and the pMD2G plasmid encodes the vesicular stomatitis virus surface glycoprotein (VSVG), which is required for assembly and the budding process of virus. These pseudotyped virions have the ability of infecting a broad spectrum of cell, even without the CD4 receptor (including Hela cells).

*Inhibition of HIV p24 core antigen production (HIV replication)*

Hela cells, which were used as target cells in this experiment, were seeded at a density of 6 × 10^4^ cells per well in 96-well plates. Each well was infected with 600 ng of p24 single cycle replicable HIV-1 (SCR HIV-1) virions. After 24 h of virus adsorption, cells were washed 3 times with pre-warmed DMEM to remove free virus particles. Cells were then incubated for 48 hours in a total volume of 200 μL per well of fresh medium containing various concentrations of compounds 1-8. Nevirapine (a HIV-1/2 RT inhibitor) was used as positive control. After 48 h, the p24 antigen (Ag) assay was performed on the supernatants by using a quantitative p24 ELISA method (HIV p24 ELISA, BioMerieux, France), according to the manufacturer’s protocol. The IC_50_ of compounds 1-8 was calculated according to the method described by Cheng *et al*.(6). The therapeutic index (TI) was evaluated as the ratio of CC_50_ to IC_50_.

*XTT-based cytotoxicity assay*

The cellular toxicity of compounds 1-8 in Hela cells was assessed using a cell proliferation XTT kit (Roche Diagnostics, Germany), as described previously (7). Briefly, cells were plated in duplicate in 96-well plates in the presence or absence of various concentrations of compounds 1-8. After incubation at 37°C with 5% CO2 for 3 days, 50 μL of prepared XTT mixture was added to each well. The cells were incubated for an additional 4 hours to allow the production of XTT formazan. Absorbance was measured using an ELISA plate reader (BioTek ELx800) at a test wavelength of 450 nm and a reference wavelength of 690 nm. Percent inhibition was calculated using the following formula: Inhibition (%) = [100 – (A_t_/A_s_)] × 100, where A_s_ is the absorbance of the solvent and A_t_, of the test sample, respectively. The cytotoxic concentration that resulted in a reduction of the number of viable cells by 50% (CC_50_) was calculated from dose response curves.

*Docking studies using AutoDock software*

The high resolution crystal structure of HIV protease (PDB code: 1AJX) complexed with Aha001 was retrieved from PDB Protein Data Bank and its ligand was deleted from the active site. Compound **7** was constructed on HyperChem 8.0.3 version and minimized using AMBER force field molecular mechanics and PolakRiebiere algorithm with RMS gradient = 0.01 Kcal/mol.

The receptor was kept rigid, and ligand was allowed to be flexible. Polar hydrogens and Kollman united atom partial charges were added to the individual protein atoms. A docking grid box was built with 28, 28 and 28 points in 12.79, 23.30 and 5.85 directions in the catalytic site of protein and the number of generations and maximum number of energy evaluations was placed on 100 and 2,700,000, respectively. Docking results were clustered with a root mean square deviation (RMSD) of 0.5 Å and evaluated by Pymol software.

## Results and Discussion

*Structures elucidation*

Compound 1a & 1b showed a pair of doublets in the up-field area (*δ*_H_ 0.32, *J* = 4.0 Hz and 0.55, *J* = 4.0 Hz) that develops hypothesis of cycloartane backbone of them. It should be mentioned that a pair of doublets in the up-field area in ^1^H-NMR spectrum is a well-known characteristic of cyclopropane ring of cycloartanes between natural products. Based on this hypothesis comparison of^ 13^C- NMR spectral data of cycloartane structures and Compound 1a & 1b ^13^C-NMR was performed which proved our hypothesis. 

Without pentacyclic structure, based on DEPT and broadband (BB) decoupled ^13^C-NMR, remaining carbons included three methyl groups (*δ*_C_ 18.3, 17.2 and 17.6), four methylene groups that two of them are involved in olefinic bonds (*δ*_C_ 31.5, 31.6 &110.9, 111.4), three methane groups which two of them are oxygenated (*δ*_C_ 35.9, 76.3 and 76.7), and a couple of quaternary carbons which are involved in double bonds (*δ*_C_ 147.5 and 147.7). The duplicity of most of the signals of the side chain in ^13^C-NMR spectra (*δ*_C_ 17.2 and 17.6, 31.5 and 31.6, 110.9 and 111.4, 76.3 and 76.7, 147.5 and 147.7) and intensity of them in comparison with the remaining peaks with identical multiplicity and cycloartane structure as a basic skeleton, indicated a mixture of two epimers at one of side chain’s carbon. In this way, taking one peak of each two close peaks, plus the remaining carbons of side chain, two methyl groups (2 CH_3_), two methylene groups (CH_2_ and =CH_2_), two methine groups (CH and CHO), and an olefinic quaternary carbon were involved in side chain which totally were in accordance of EI-MS *m/z *315 [M – 127]^+^ and 297 [M – H_2_O – 127] as a result of side chain fragmentation. It is crystal clear that the quaternary olefinic carbon is linked to an olefinic methylene group and therefore, the ^1^H-NMR signal for this methylene group has appeared as a doublet in the downfield region (*δ*_H_ 4.88, *J* = 1.2 Hz and 4.93, *J* = 1.2 Hz). A triplet peak at *δ*_H_ 4.02 (*J* = 6.4 Hz) with the integration equals to one hydrogen, demonstrated neighboring oxygenated methine to an aliphatic CH_2_ in one side and quaternary carbon in another side. A singlet at *δ*_H_ 1.72 cleared that one of methyl groups is connected to quaternary carbon while another methyl linked to a methine because of its doublet multiplicity (*δ*_H_ 0.88, *J* = 5.6). Finally, considering these data and literature (8) compound **1a** was determined as (24*R*)-cycloart-25-ene-3*β*,24-diol which was mixed with its C-24 epimer compound 1b; (24*S*)-cycloart-25-ene-3*β*,24-diol.

Cycloartane skeleton of compound 2 was confirmed by a pair of doublets in the up-field area (*δ*_H_ 0.26, *J* = 4.0 Hz and 0.49, *J* = 4.0 Hz) and comparison of ^13^C- NMR spectrum between compound 2 and 1a. In addition to the carbons which are involved in cycloartane skeleton, ^13^C-NMR (BB and DEPT) showed three methyl groups, a methylene group and three methine group that two of them are olefinic (*δ*_C_ 124.6 and 138.3) and the third one is an oxygenated quaternary carbon (*δ*_C_ 70.7). The presence of These additional carbons is in agreement with the ESI-MS *m/z *at 315 [M – 127]^+^ and 297 [M – H_2_O – 127]^+ ^as the results of cycloartane side chain fragmentations. Taking these features into consideration and reviewing literatures (8, 9), compound 2 was determined as cycloart-23-ene-3*β*,25-diol.

Existence of cycloartane structure in the backbone of compound 3 was revealed by the presence of a pair of doublets in ^1^H-NMR spectrum (*δ*_H_ 0.33, *J* = 4.0 Hz and 0.53, *J* = 4.0 Hz) and comparison of compounds 3 and 1a ^13^C-NMR spectra. Moreover, based on BB decoupled ^13^C-NMR spectrum and DEPT, side chain contained three methyl groups, two methylene groups, one oxygenated methine group and an oxygenated quaternary carbon in accordance of EI-MS *m/z* at 316 [M – 145]^+^ and 297 [M – H_2_O – 145]^+^ that are side chain fragmentation results. Two singlet methyl signals at *δ*_H_ 1.16 and 1.22 suggested the linkage of two methyl groups and oxygenated quaternary carbon and the third methyl has a doublet at *δ*_H_ 0.89 (*J* = 5.6 Hz) which points to neighboring methyl to methine. Finally, reviewing the literatures (8, 10) and considering these data, compound 3 was determined as cycloart-3*β*,24,25-triol.

Compound 4 exhibited a positive HRESI-MS pseudo-molecular ion [M + H]^+^ at *m/z* 457.3623 suggesting its molecular formula as C_30_H_48_O_3_. Broadband decoupled ^13^C-NMR and DEPT spectra, showed the presence of an aliphatic backbone which has a double bond consisting of a methine group and a quaternary carbon (*δ*_C_ 122.6 and 143.6 respectively). Furthermore, a carbonyl group (*δ*_C_ 183.2) was identified that along with the loss of *m/z* = 45 at EI-MS indicated the presence of a carboxylic acid functional group. The presence of a carboxylic group and a double bond, taking together with seven degrees of unsaturation, supported pentacyclic structure of compound **4**. Olefinic proton was clear with a triplet (*δ*_H_ 5.28, *J *= 3.4 Hz) and seven angular methyl singlets at *δ*_H_ 0.75, 0.77, 0.90, 0.91, 0.93, 0.99 and 1.13 were also detected. In addition, two signals at *δ*_H_ 3.22 (1H, *dd*, *J* = 11.0, 4.6 Hz) and 2.82 (1H, *dd*, *J* = 13.8, 4.2 Hz) represented an oxygenated and allylic protons, respectively. These data suggested oleanene skeleton in agreement with EI-MS *m/z* 248 and 207, which are diagnostically important peaks of an olean-12-ene structure that has a carboxylic functional group at C-17, as a result of retro Diels-Alder cleavage of ring C (11). According to the previously reports for oleanolic acid (12, 13), the hydroxyl group on position 3 has a beta configuration and therefore the carbinolic hydrogen (H-3) has an alpha configuration. The ^1^H-NMR signal for this hydrogen has appeared as a doublet of doublets (1H, *J* = 11.0, 4.6 Hz) at *δ*_H_ 3.22. Eventually, the foregoing characteristics as well as published papers (12, 13), determined that compound **4** is oleanolic acid.

*Anti HIV activity*

**Figure 3:**
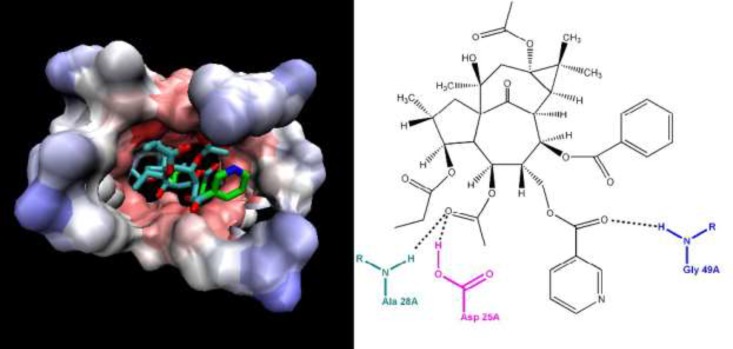
Ligand-receptor binding model for compound **7 **and HIV-1 protease (pdb 1AJX

Anti-human immunodeﬁciency virus-1 (HIV-1) activity of compounds **1-4 **and previously reported ingenoid diterpenes from *E. erythradenia* was evaluated (Table 3). The four ingenoid diterpenes had been identified previously and their pro-apoptotic effect has been established (14). Based on literatures, it is known that oleanolic acid (15, 16) and cycloartanes (17) partially and diterpenes especially are cytotoxic which our results confirm that. But compound **7** showed significant anti HIV activity (IC_50_ = 0.008 μM, CC_50_ = 3.264 μM, TI = 380.64) and higher therapeutic index than nevirapine, a non-nucleoside reverse transcriptase inhibitor (NNRTI) used to treat HIV-1 infection and AIDS. Ligand protein binding studies between compound **7** and HIV-1 protease (pdb 1AJX) demonstrated three hydrogen bond between Asp 25A, Asp 28A and Gly 49A of protease active site and compound **7** with a distance less than 3 A° that can be responsible for the observed anti HIV-1 activity (Figure 3).

**Table 3:** Anti- HIV activity of terpenoid compounds from *E. erythradenia*

Compound	Anti HIV activity	Cytotoxicity	Therapeutic index
	IC_50_ (μM)^a^	CC_50_ (μM)^b^	TI (CC_50_/IC_50_)
*1a & 1b*	0.003	0.014	4.67
*2*	0.006	0.022	3.67
*3*	0.002	0.011	5.5
*4*	0.009	0.022	2.44
*5*	0.036	0.004	0.11
*6*	0.007	0.084	11.75
*7*	0.008	3.264	380.64
*8*	0.044	0.168	26.68
*Nevirapine*	< 0.064	6.74	> 105.31

a IC_50_ = Concentration causing 50% HIV activity inhibition.

b CC_50_ = Concentration causing 50% cellular cytotoxicity.

## References

[1] Jassbi AR (2006). Chemistry and biological activity of secondary metabolites in Euphorbia from Iran. Phytochemistry.

[2] Mozaffarian V (1994). Plant Systematics.

[3] Haba H, Lavaud C, Harkat H, Alabdul Magid A, Marcourt L, Benkhaled M (2007). Diterpenoids and triterpenoids from Euphorbia guyoniana. Phytochemistry.

[4] Zabihollahi R, Sadat SM, Vahabpour R, Aghasadeghi MR, Memarnejadian A, Ghazanfari T, Salehi M, Rezaei A, Azadmanesh K (2011). Development of single-cycle replicable human immunodeficiency virus 1 mutants. Acta Virol.

[5] Sadat S, Zabihollahi R, Vahabpour R, Azadmanesh K, Javadi F, Siadat S, Memarnejadian A, Parivar K, Khanahmad Shahreza H, Arabi Mianroodi R Designing and biological evaluation of single-cycle replicable HIV-1 system as a potential vaccine strategy. Proceedings of the 20th European Congress of Clinical Microbiology and Infectious Diseases; 2010 Apr 10-13.

[6] Cheng HY, Lin CC, Lin TC (2002). Antiherpes simplex virus type 2 activity of casuarinin from the bark of Terminalia arjuna Linn. Antivir. Res.

[7] Cheng PW, Ng LT, Chiang LC, Lin CC (2006). Antiviral effects of saikosaponins on human coronavirus 229E in vitro. Clin. Exp. Pharmacol. Physiol.

[8] Della Greca M, Fiorentino A, Monaco P, Previtera L (1994). Cycloartane triterpenes from Juncus effusus. Phytochemistry.

[9] Shamsabadipour S, Ghannadian M, Saeedi H, Rahimnejad MR, Mohammadi-Kamalabadi M, Ayatollahi SM, Salimzadeh L (2013). Triterpenes and steroids from Euphorbia denticulate Lam with Anti-Herpes Symplex virus activity. Iran. J. Pharm. Res.

[10] Inada  A, Ohtsuki S, Sorano T, Murata H, Inatomi Y, Darnaedi D, Nakanishi T (1997). Cycloartane triterpenoids from Aglaia harmsiana. Phytochemistry.

[11] Ayatollahi AM, Ghanadian M, Afsharypour S, Abdella OM, Mirzai M, Askari G (2011). Pentacyclic triterpenes in Euphorbia microsciadia with their t-cell proliferation activity. Iran. J. Pharm. Res.

[12] Shin SJ, Park CE, Baek NI, Chung IN, Park CH (2009). Betulinic and oleanolic acids isolated from Forsythia suspensa Vahl inhibit urease activity of Helicobacter pylori. Biotech. Bioprocess. Eng.

[13] Seebacher W, Simic N, Weis R, Saf R, Kunert O (2003). Complete assignments of 1H and 13C NMR resonances of oleanolic acid, 18α‐oleanolic acid, ursolic acid and their 11‐oxo derivatives. Magn. Reson. Chem.

[14] Zarei SM, Ayatollahi AM, Ghanadian M, Kobarfard F, Aghaei M, Choudhary MI, Fallahian F (2013). Unusual ingenoids from Euphorbia erythradenia Bioss with pro-apoptotic effects. Fitoterapia.

[15] Li J, Guo W-J, Yang Q-Y (2002). Effects of ursolic acid and oleanolic acid on human colon carcinoma cell line HCT15. World J. Gastroenterol.

[16] Fernandes J, Castilho RO, da Costa MR, Wagner-Souza K, Coelho Kaplan MA, Gattass CR (2003). Pentacyclic triterpenes from Chrysobalanaceae species: cytotoxicity on multidrug resistant and sensitive leukemia cell lines. Cancer. Lett.

[17] Banskota AH, Tezuka Y, Phung LK, Tran KQ, Saiki I, Miwa Y, Taga T, Kadota S (1998). Cytotoxic cycloartane-type triterpenes from Combretum quadrangulare. Bioorg. Med. Chem. Lett.

